# Supplementary material for: Systematic Comparison of High-throughput Single-Cell and Single-Nucleus Transcriptomes during Cardiomyocyte Differentiation
Source: Sci Rep. 2020 Jan 30;10:1535. doi: 10.1038/s41598-020-58327-6 (PMC6992778; doi:10.1038/s41598-020-58327-6)
Supplement: Supplementary file 1 — Supplementary information. [file 41598_2020_58327_MOESM1_ESM.docx]

Systematic Comparison of High-throughput Single-Cell and Single-Nucleus Transcriptomes during Cardiomyocyte Differentiation

Alan Selewa^1,4^, Ryan Dohn^1^, Heather Eckart^1^, Stephanie Lozano^1^, Bingqing Xie^1^, Eric Gauchat^1,4^, Reem Elorbany^2^, Katherine Rhodes^2^, Jonathan Burnett^2^, Yoav Gilad^1,2^, Sebastian Pott^2*^, Anindita Basu^1,3*^

**Supplementary Methods**

Species-mixing and single-cell specificity

For the Drop-seq experiment on biological replicate #1, chimpanzee iPSCs^20^ were mixed with human iPSC-derived CMs from day 7 of the differentiation time-point, in order to assess the frequency of doublets during cell encapsulation. We used chimpanzee cells for the species mixing as these cells were grown using identical conditions as the human cells. The alignment protocol was adjusted so that each read was aligned to both the human genome (GRCh38) and the chimp genome (panTro5) separately. For each cell that passed quality control, we counted the number of reads that aligned exclusively or with a better score to the genome of one of the species (Figure S1). We then used the ratio of these counts as a ‘species-specificity’ score for each cell. We found only a small number of cells with scores that could suggest mixing of cells from human and chimp (< 5%), similar to previously reported estimates^5^. Cells with intermediate scores had typically lower read counts and were thus removed by filtering based on read depth. We only kept cells with a specificity score above 0.6 yielding ~739 cells. In agreement with our assignment, > 99% of these cells were associated with clusters that we identified as CMs while none were associated with iPSC clusters.


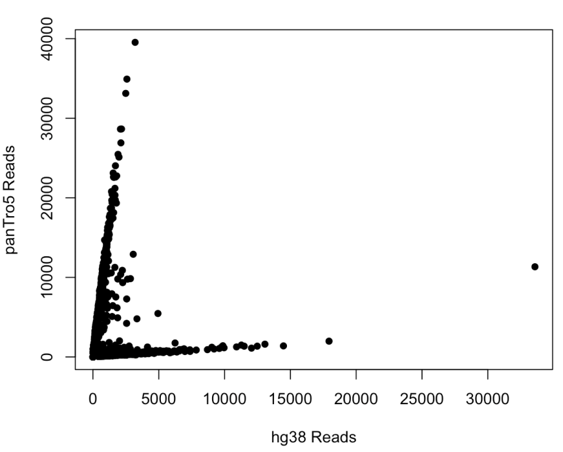


**Figure S1:** Scatterplot of number of reads assigned to hg38 vs panTro5 for each cell in Drop-seq day 7, cell line #1 as part of a species-mixing experiment using human iPSC derived cardiomyocytes and chimpanzee iPSCs.


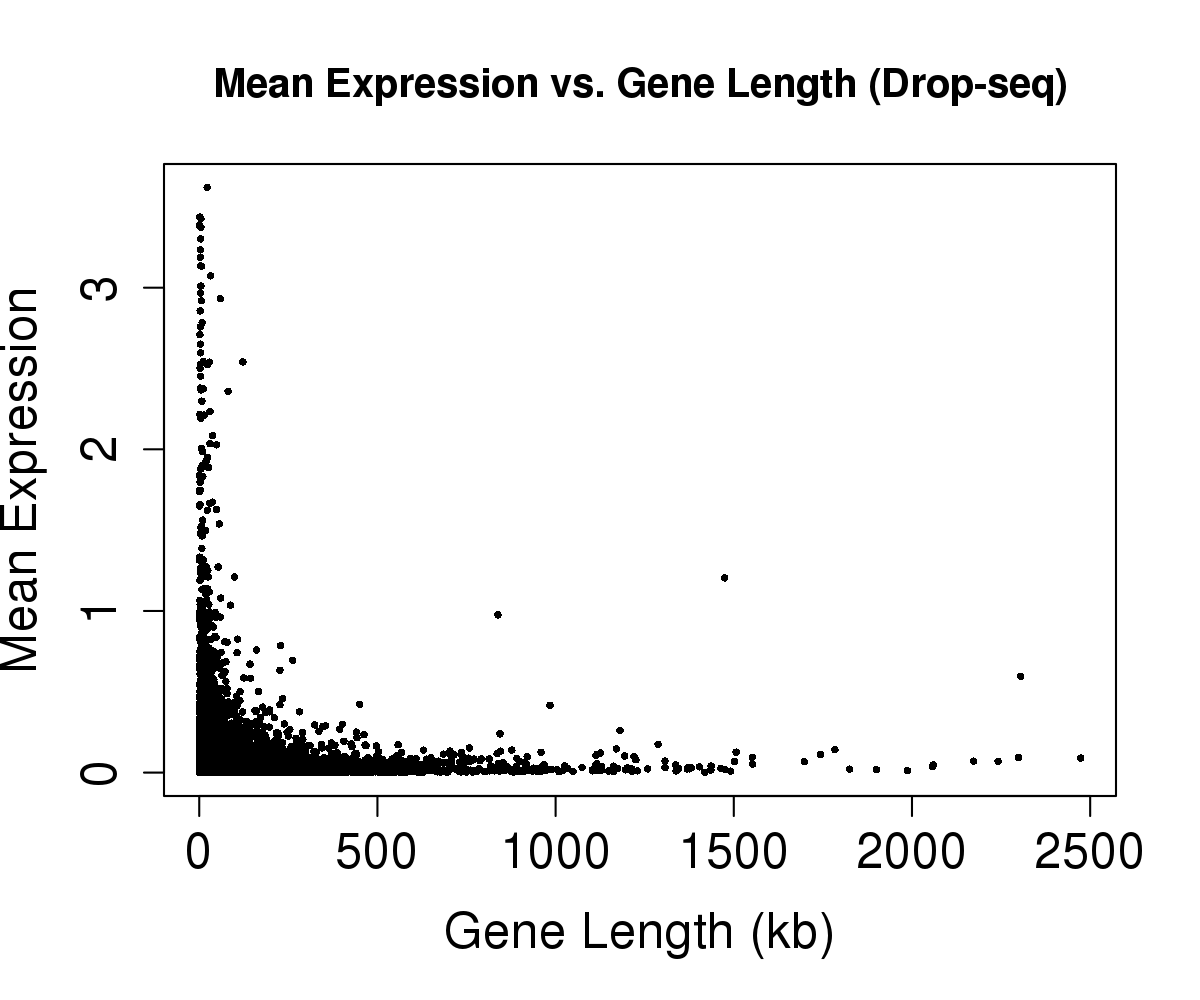

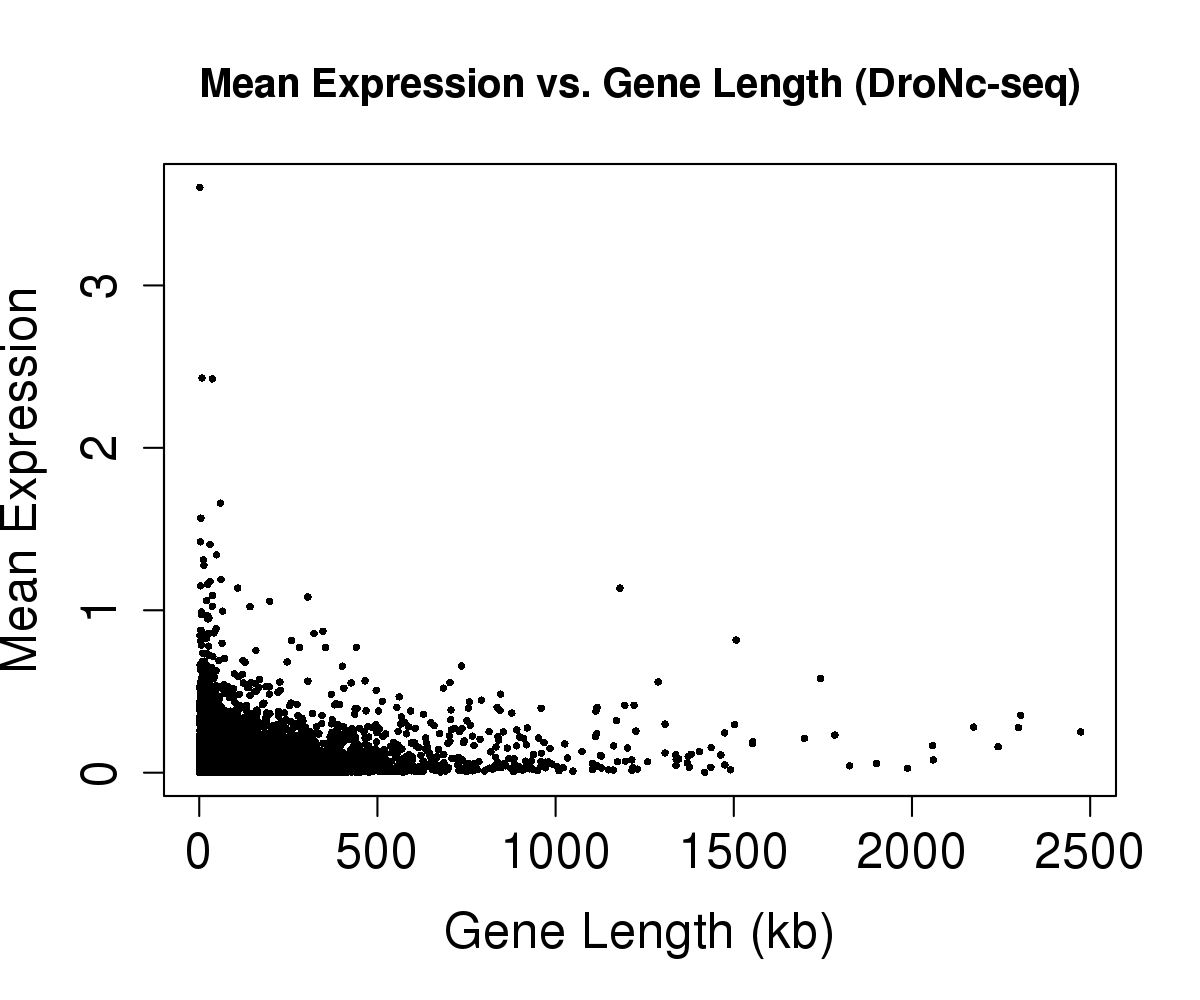


**Figure S2:** Mean expression (log) vs. gene length for Drop-seq (left) and DroNc-seq (right).


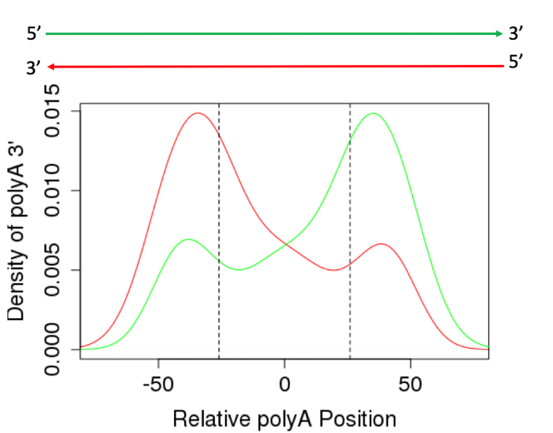


**Figure S3:** Density curves of the position of polyA at the 3’ end. Green and red curves represent reads mapping to the forward and reserve direction, respectively. The dashed line represents the average read length.


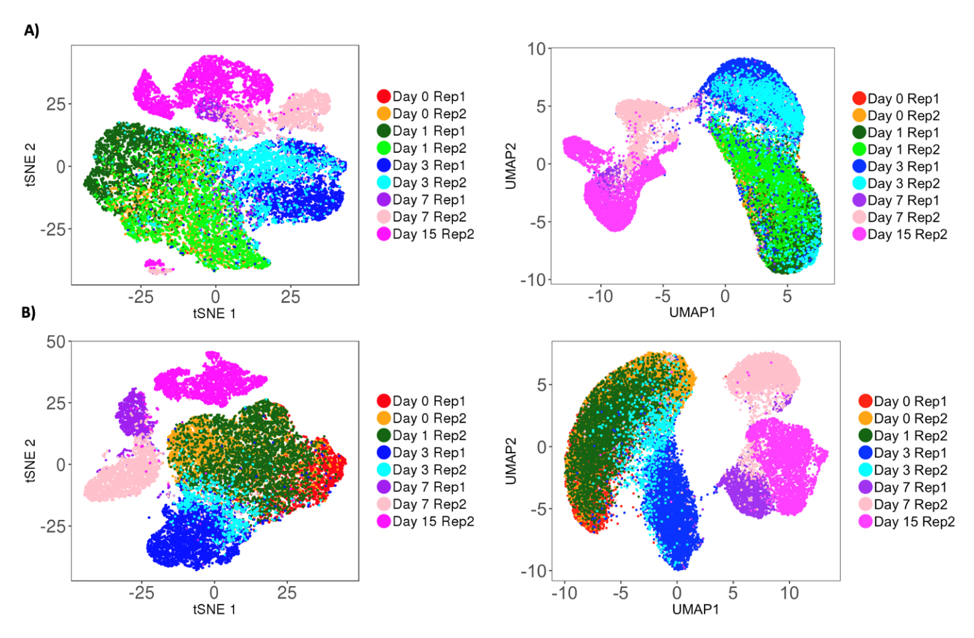


**Figure S4:** Dimensionality reduction for A) Drop-seq and B) DroNc-seq using tSNE (left) and UMAP (right). Color represents the differentiation time point.


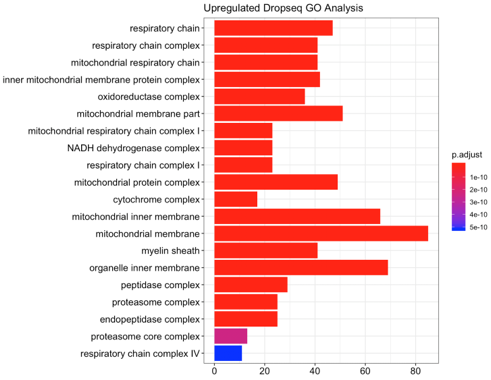

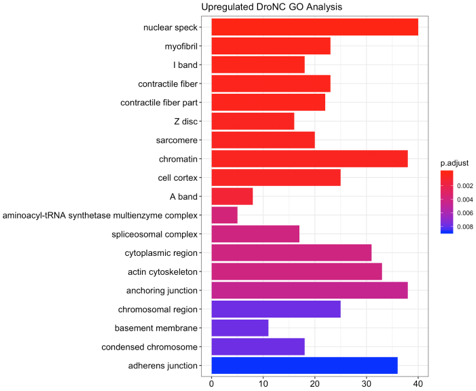


**Figure S5:** Gene enrichment analysis on differentially expressed genes between Drop-seq and DroNc-seq.


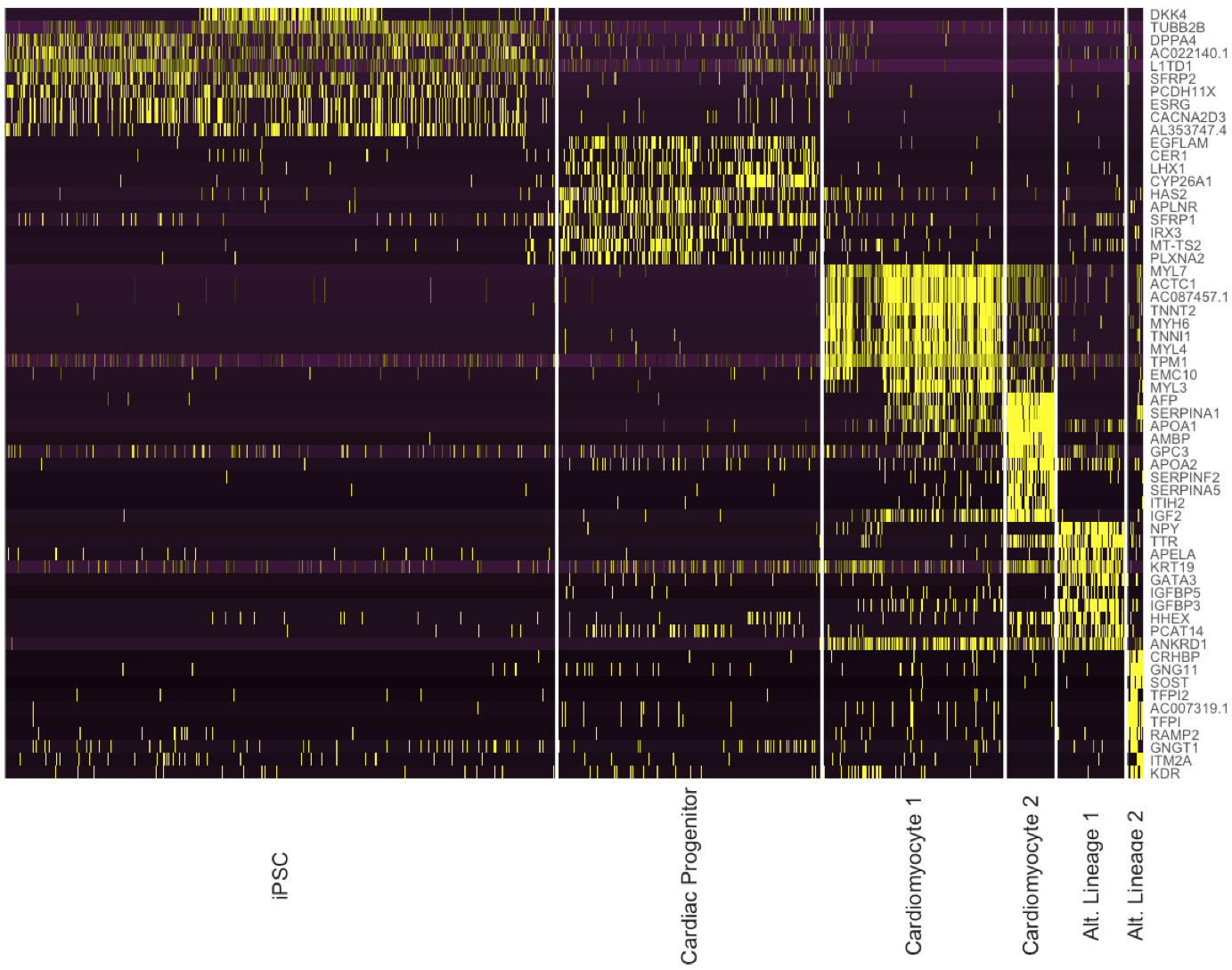


**Figure S6:** Heatmap of expression values of top 10 differentially expressed genes in each cell type cluster for Drop-seq.


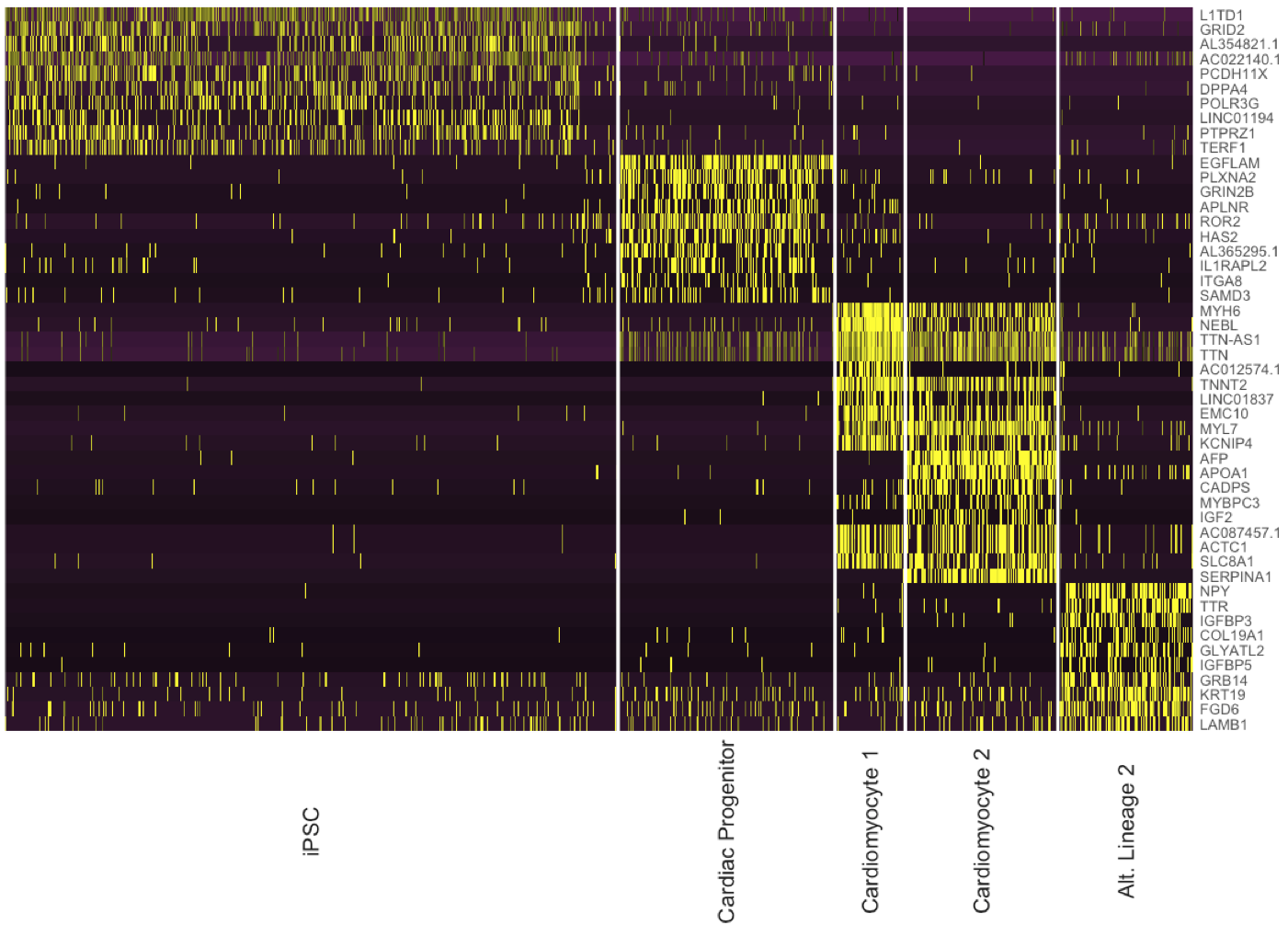


**Figure S7:** Heatmap of expression values of top 10 differentially expressed genes in each cell type cluster for DroNc-seq.

Table S1: Breakdown of cell types and associated genes discovered in Drop-seq and DroNc-seq

| **Markers** | **Cell type** | **Prevalence (Drop-seq)** | **Prevalence (DroNc-seq)** | **Drop-seq Only Genes (top 5)** | **DroNc-seq Only**  **Genes (top 5)** |
| --- | --- | --- | --- | --- | --- |
| DPPA4 | iPSC | 48.9% | 52% | SFRP2, AC025465.1, ESRG, CACNAD2D3, BDNF-AS | RIMS2, RPL8,  GOLGA4, EIF4A2,  SET |
| EOMES  APLNR | Cardiac  Progenitor | 23.3% | 18.2% | CER1, LHX1, CYP26A1, IRX3, MT-TS2 | GRIB2B, AL3365295.1, IL1RAPL2, KCNQ5, NRX3 |
| MYH6  TNNT2 | Cardiomyocyte 1 | 16.1% | 5.6% | MYL3, NPPA-AS1, NPPA, ACTN2, TNNC1 | AC012574.1, AC105233.5, MYO1D, ARHGAP42, CDK14 |
| MYH6  TNNT2  AFP  SERPINA1 | Cardiomyocyte 2 | 4.2% | 12.7% | AMBP, APOA2, SERPINF2, ITIH2, SERPINA5 | KCNH7, ERBB4, ZBTB20, NRG3, KCNQ5 |
| TTR  FOXA2 | Alternative Lineage 1 | 5.9% | 11.3% | GATA3, S100A14, HHEX, FLIRT3, EPSTIL1 | EWSR1, PTBP2, ZMYM2, LUC7L, LINC01876 |
| CD34  SCARF1  FLT1 | Alternative Lineage 2 | 1.4% | 0% | CRHBP, GNG11, SOST, TFPI2, AC007319.1 | None |


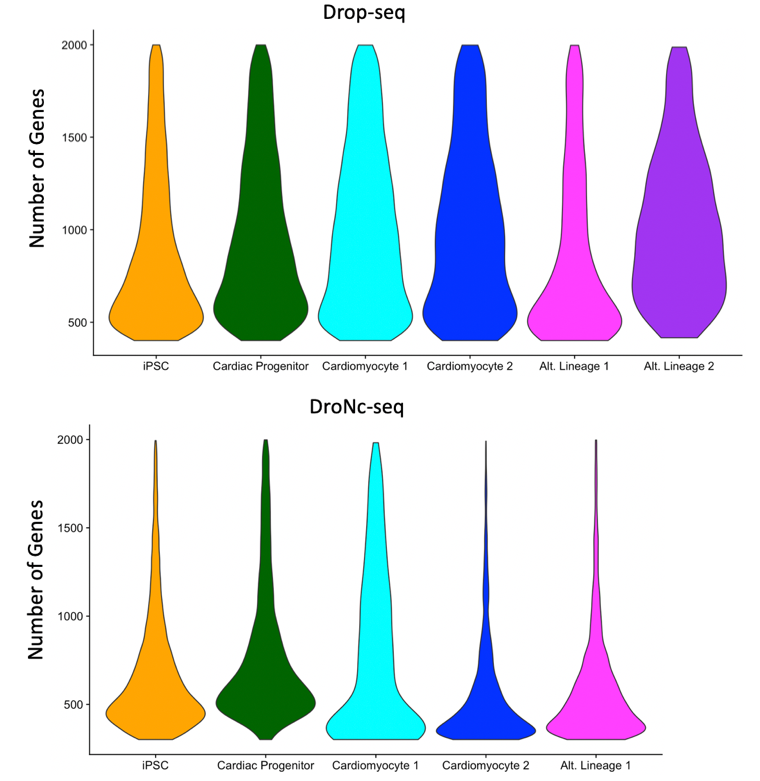


**Figure S8:** Violin plots representing the of number of genes in each cluster for Drop-seq (top) and DroNc-seq (bottom).


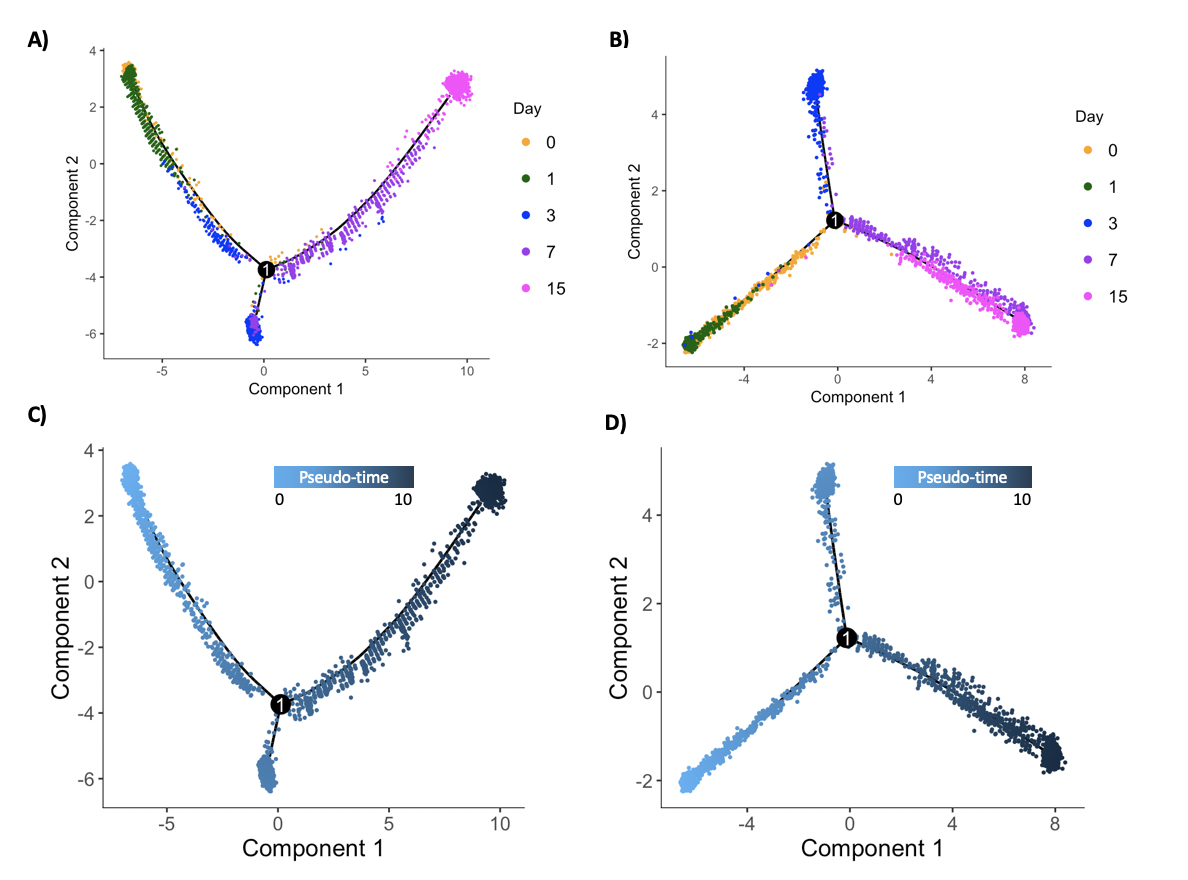


**Figure S9:** Cell differentiation trajectories constructed from Drop-seq (left), and DroNc-seq (right) using Monocle. Each differentiation time-point sampled is labelled by the same color in both techniques. A, B) uses the time-point as color, and C, D) shows the inferred pseudo-time as the color.


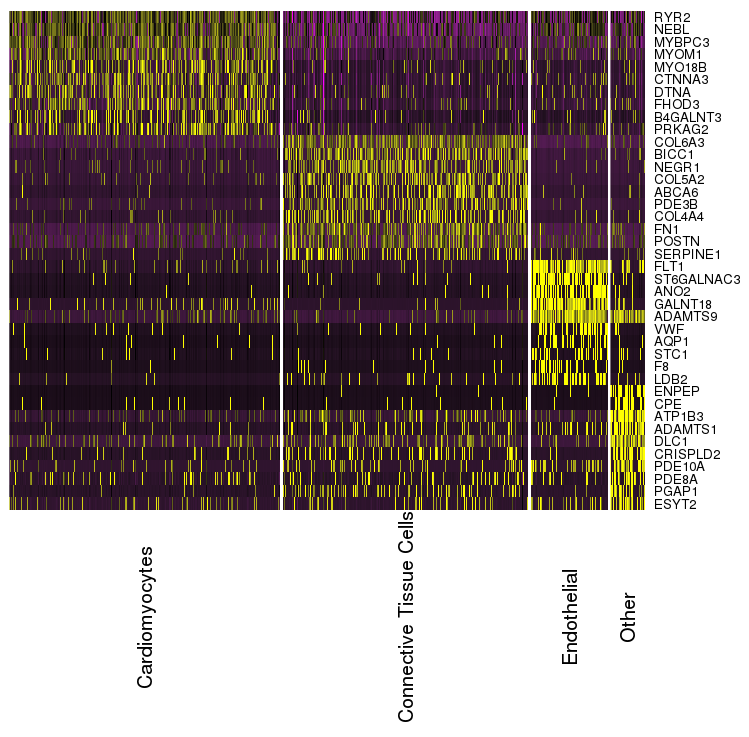


**Figure S10:** Top 10 upregulated genes identified in each cell type cluster using DroNc-seq on primary tissue from archived adult human heart.


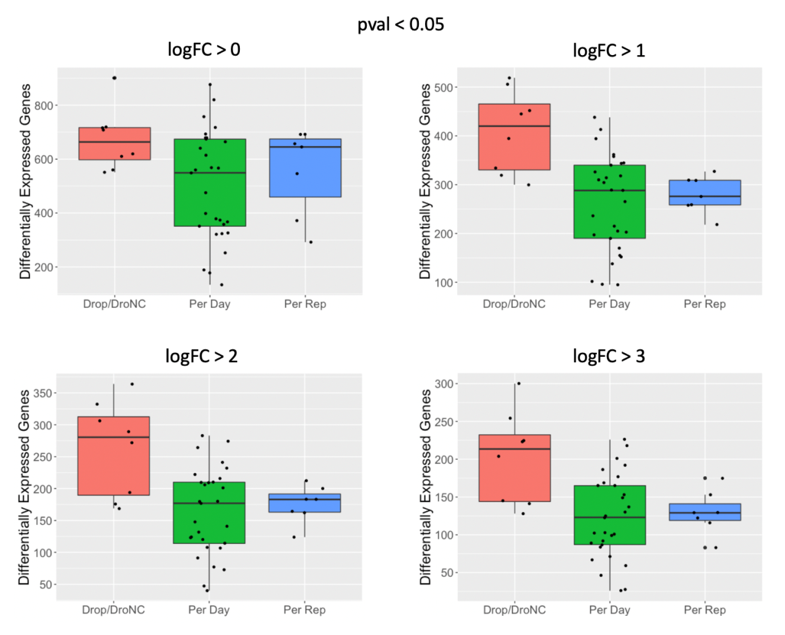


**Figure S11:** Differential expression analysis across time-points, cell-lines (biological replicates), and across Drop-seq and DroNc-seq using different thresholds for log-fold-change. All genes shown have adjusted p-value < 0.05.


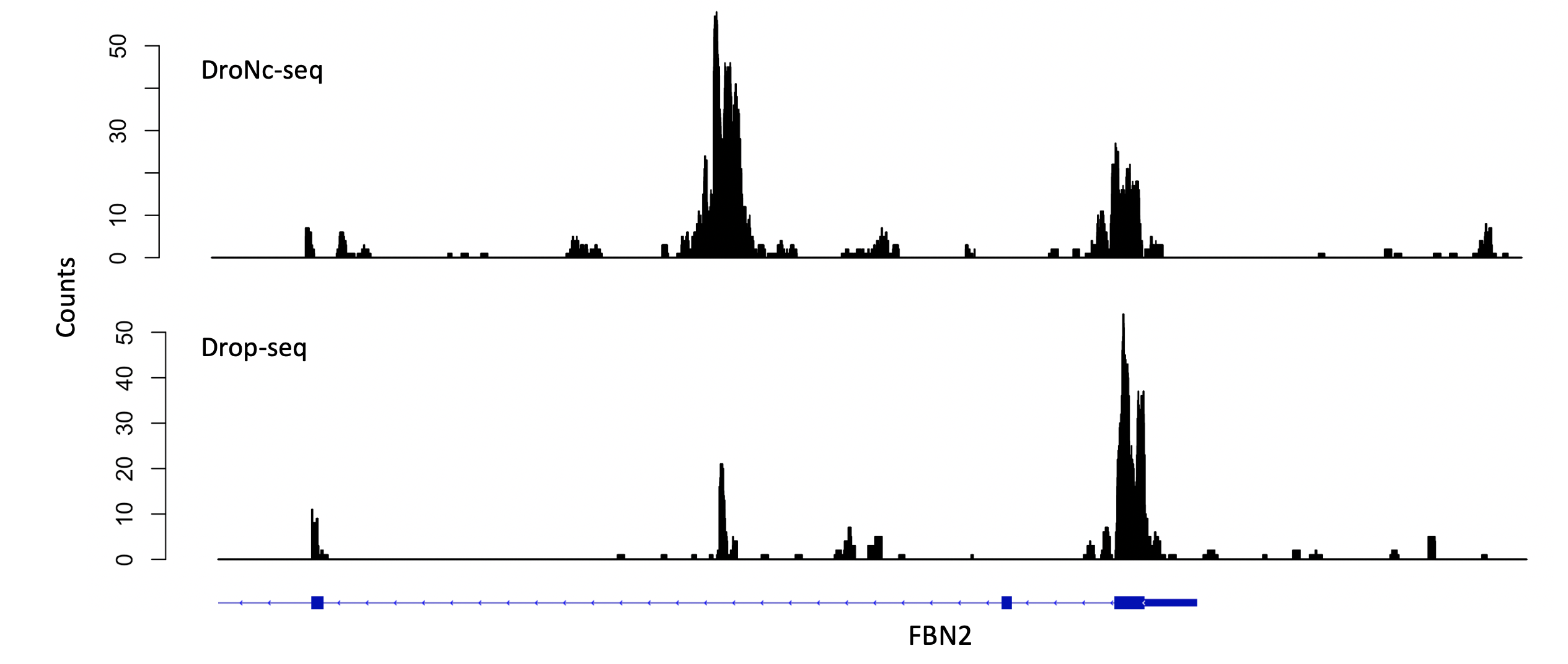


**Figure S12:** Read count distribution across example loci FBN2 within DroNc-seq and Drop-seq data.


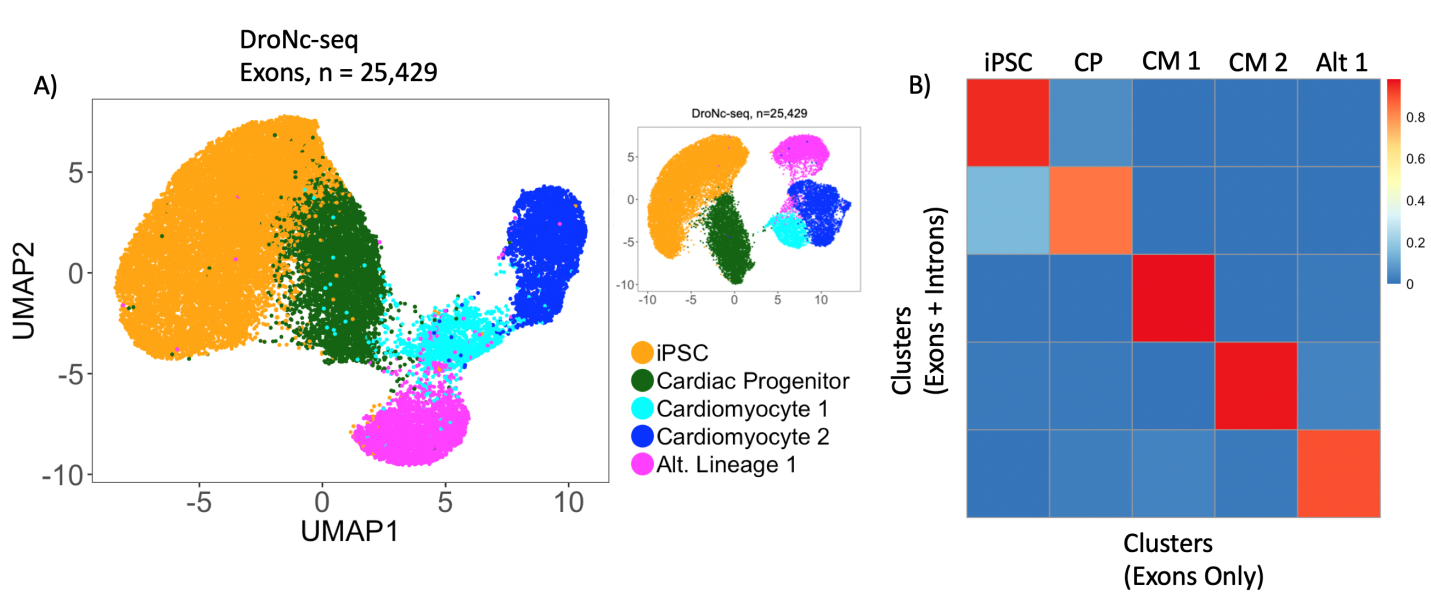


**Figure S13:** A) UMAP of DroNc-seq counts generated with exons only. Inset shows the UMAP results with exons and introns. B) Confusion matrix representing the proportion of cells shared between clusters found in exons + introns data and exons-only data, for DroNc-seq.


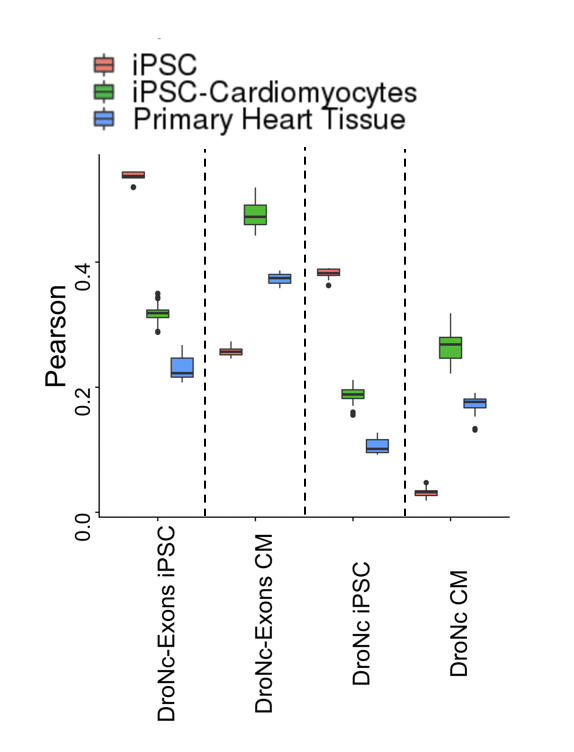


**Figure S14:** Pearson correlation with bulk samples compared between exon-only and intron/exon generated counts in DroNc-seq.


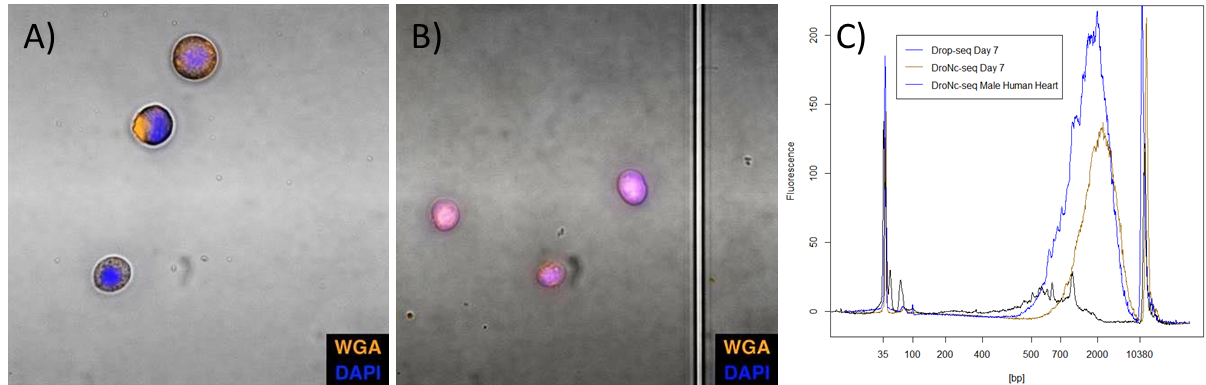


**Figure S15**: Experimental quality control metrics. Images of Day 1 of differentiation of human iPSC derived cardiomyocyte (iPSC-CM) cells- A) and nuclei- B) stained with DAPI and WGA; C) BioAnalyzer traces of WTA product from Drop-seq on iPSC-CM Day 7, DroNc-seq on iPSC-CM Day 7, and DroNc-seq on archived adult male heart tissue.

In order to adopt a criterion for choosing the resolution, we turned to the SC3 stability index (Kiselev 2017) as defined in the equation below. The stability index compares a cluster at a particular resolution with all other clusters at all other resolutions. If the cluster being evaluated undergoes splitting for all changes of resolution, then it will be evaluated as relatively unstable. The stability index ranges from 0 to 1 denoting less stable to more stable, respectively.

$$s\left( k,r \right)=\frac{1}{\left| L \right|}\sum_{l\in L} \sum_{j\in N_{l}} \frac{c\left( k,r \right)\cap c\left( l,j \right)}{c\left( l,j \right) N_{l}^{2}}$$

We ran the Seurat FindClusters algorithm for a range of resolutions from 0.01 (2 clusters) to 0.4 (12 clusters), and computed the stability index for each cluster and each resolution (see Figure S16 below). At each resolution, we took the mean stability across all clusters. The resulting stability profiles are shown below for DroNc-seq and Drop-seq. We used kernel regression in R (ksmooth function) to fit a line to the data. From these results, we see that mean stability is maximum near resolutions of 0.11 and 0.14 (red dashed line) for DroNc-seq and Drop-seq leading to 5 and 6 clusters, respectively. These values are close to the value of 0.13 that we originally chose. Importantly, the small differences in resolution parameters do not lead to any changes in number of clusters discovered and cell assignments to clusters.


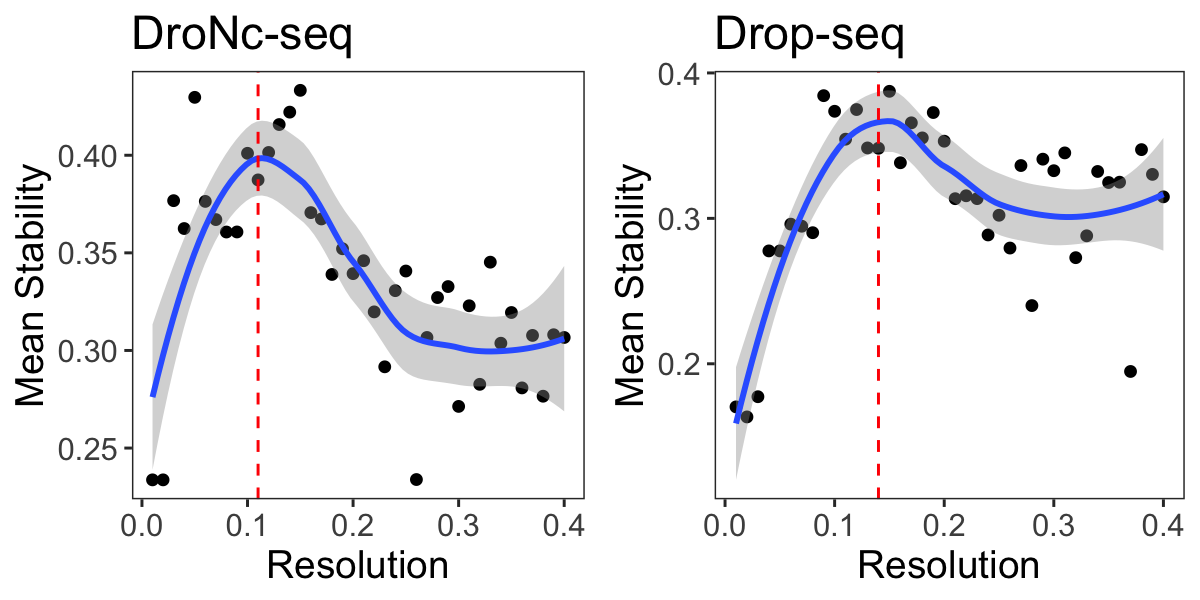


**Figure S16:** Cluster stability index computed for a range of resolution parameters. The red vertical dashed line represents the resolution at which the mean stability is maximum.

Table S2: Viability of harvested cells from each iPSC-CMs differentiation time-point

| Time Point | Date | Viability |
| --- | --- | --- |
| Time Course 1 Day 0 | 11/16/2017 | 70% |
| Time Course 1 Day 1 | 11/15/2017 | 50% |
| Time Course 1 Day 3 | 11/17/2017 | 80% |
| Time Course 1 Day 7 | 11/21/2017 | 60% |
| Time Course 2 Day 0 | 1/22/2018 | 60% |
| Time Course 2 Day 1 | 1/23/2018 | 80% |
| Time Course 2 Day 3 | 1/25/2018 | 80% |
| Time Course 2 Day 7 | 1/29/2018 | 90% |
| Time Course 2 Day 15 | 2/6/2018 | 55% |
